# Organizational Justice and the Shortage of Nurses in Medical & Educational Hospitals, in Urmia-2014

**DOI:** 10.5539/gjhs.v8n2p99

**Published:** 2015-06-11

**Authors:** Heidar Sharifi Fathabad, Abbas Yazdanpanah, Somayeh Hessam, Elham Ehsani Chimeh, Siamak Aghlmand

**Affiliations:** 1 Research School, Department of Healthcare Management, Marvdasht Branch, Islamic Azad University, Marvdasht, Iran; 2 Department of Healthcare Management, Marvdasht Branch, Islamic Azad University, Marvdasht, Iran; 3 Islamic Azad University, Shiraz, Department of Health Services Administration, Shiraz, Iran; 4 Human Resource for Health Office, Ministry of Health and Medical Education, Tehran, Iran; 5 Social Determinant of Health Research Center, School of Public Health, Urmia University of Medical Sciences, Urmia. Iran

**Keywords:** organizational justice, distributive justice, procedural justice, interactive justice, turnover, nurse, leaving job

## Abstract

**Objective:**

One of the most important reasons of turnover is perceptions of organizational justice. The purpose of this study was to investigate the effect of perceived organizational justice and its components on turnover intentions of nurses in hospitals of Urmia University of Medical Sciences.

**Methods:**

This cross-sectional study was among nurses. 310 samples were estimated according to Morgan Table. Two valid and reliable questionnaires of turnover and organizational justice were used. Data analysis was performed using the software SPSS20. Using the Kolmogorov-Smirnov test, the normality and relationship between variables with Pearson and Spearman correlation test were analyzed.

**Results:**

Most people were married and aged between 26 and 35 years, BA and were hired with contraction. The mean score of organizational justice variable was 2.59. The highest average was the interactional justice variable (2.81) and then Procedural fairness variable (2.75) and distributive justices (2.03) were, respectively. The mean range of turnover variable was 3.10. The results showed weak and negative relationship between various dimensions of organizational justice and turnover in nurses.

**Conclusion:**

Organizational justice and turnover had inverse relationship with each other. Therefore how much organizational justice in the organization is more; employees tend to stay more. Finally, suggestions for improvement of justice proposed.

## 1. Introduction

Due to the high cost of training and maintenance of human resources, organizations always have this fear of losing their human capital losses, because the loss of valuable forces, resulted in a loss of skills and experience that were obtained over the years (Cascio, 1991; Hom & Griffeth, 1994). However, today maintaining the professionals is one of the challenges that rose in all organizations, regardless of type or geographical location (Arnold, 2005). Contrary to real job leaving, tend to leave the job, is not clear. Desires, expressed in terms of a particular behavior of interest (Perez, 2008). Tend to leave the job is defined thinking mentally to leave the job in a period of time and it is a most important prerequisite of real job leaving (Sousa-Poza & Henneberger, 2004). Leaving the profession is also influenced and increased by factors such as management strategies, shortage of human resources for health, salaries, stress and stressors (Erlen, 2001).

Studies have shown that a shortage of nurses because of leaving the profession has a great influence on the quality of care because at the time of service, quality of care has been influenced by quantity (Corley, Minick, Elswick, & Jacobs, 2005) And because of that negative effects such as increased waiting times to receive care were happened and will lead to increase the complains (Meltzer & Huckabay, 2004). On the other hand, shortage of nurses results in shortage of human resource for health and consequently increasing the average age of remain nurses (Andrews & Dziegielewski, 2005). Statistics shown that more than 40 percent of the formal human resources for health, in US hospitals are over 50 years of old (Norman et al., 2004). This force will retire within a shorter time and leave the workforce crisis in health care environments (Andrews & Dziegielewski, 2005). However, the lack of information on the turnover of nurses, make difficult to understanding the effects of it and also nursing complexity for nurses and health administrators.

Several strategies have been proposed to prevent the process of leaving the profession Such that it is possible to create a positive working environment, recognizing the need for flexibility and balance staff working condition with his individual life (Norman et al., 2004). One of the factors in the context is organizational justice that directly affects people’s willingness to leave.

Organizational justice is an important means of motivation in organizational behavior. Justice is a broad and multifaceted concept and discipline and in different branches of a philosophical concept with the meaning of non-discrimination and fair differences (Mardani Hamooleh & Heydari, 2009). Organizational justice is used to describe the perception of a group and individual fairness of the behavior conducted by an organization and their behavioral responses to such perception (Aryee, Budhwar, & Chen, 2002).

Given the financial and human costs of nurses’ turnover in healthcare organizations, projection of it is necessary for preventing costly and unnecessary job leaving (Barlow & Zangaro, 2010). Because many studies have shown that a direct relationship between predicted turnover and actual turnover of nurses (Brooten, Youngblut, Kutcher, & Bobo, 2004; Lucas, Atwood, & Hagaman, 1993; Shader, Broome, Broome, West, & Nash, 2001), though The purpose of this paper is to examine the relationship between a desire to leave in nurses and their perceptions of organizational justice in the educational hospitals of Urmia. According to the study along the confidentiality of information of nurses and hospitals, research ethics were concidered and there is no conflict of interest.

## 2. Method

The research population of descriptive correlational study is contract, agreement or formal employed nurses, with at least 1 year of experience in hospitals of Urmia University of Medical Sciences (like hospitals, Motahari martyr, Imam Khomeini, Ayatollah Taleghani and Sydalshhda). Sample size was estimated 310 person through Morgan table and these samples was divided in proportion to the number of employees between 5 hospitals those are displayed in Table 1. Nurses among 14 hospital wards were selected and filled questionnaire.

**Table 1:** Number of samples in proportion to the number of nurses

*Samples*	*Nurses*	*Hospital Name*
117	354	Imam Khomeini
67	205	Motahari Martyr
61	187	Taleghani
53	163	Sydalshohada
12	36	Razi
310	945	Sum

Two questionnaires (standard questionnaire with 20 questions of organizational justice by Niehoff and Moorman (1993)) and is a valid and reliable questionnaire 3 questions of turnover from Golparvar study (2010) was used for data collection. The first questionnaire questions of distributive justice 1 to 5, 6 to 11 questions related to procedural justice and interactional justice Questions 12 to 20 are in the range of options for both questionnaires “totally disagree, disagree, no comment, I agree, I quite agree”, respectively. Nurse’s profile (gender, age, marital status, type of employment, work experience and organizational position) were also included.

The data were analyzed using SPSS version 20 using descriptive statistics and analysis statistics. Before using the analysis statistics, using the Kolmogorov-Smirnov test, normality of variables was examined. To examine this hypothesis, Spearman and Pearson correlation coefficient test were used.

## 3. Results

334 demographic data of the nurses is shown in Figure 1. Of the nurses in the study, 83% male, 76% married, 79% are aged between 26 and 45 years, about 96 percent of bachelor’s, 53 percent of them were employed in the formal and contractual contract. About 90% of the nurses had nursery education field and 46% had 10 years’ experience (Table 2). The mean score of organizational justice 2.59±0.04 and tend to leave out the 3.1±0.07 respectively.

**Table 2:** Demographic characteristics of nurses

Employment Type	Sex	Marital status	Age	Side	Education	Years of service
**Corporate 8.1%**	Male 83.2%	Single 24%	Under 25 years 14.6%	Nurse 89.8%	BA 95.8%	Less than 10 years 54.2%
**Contractual 38.9 %**	Female 16.8%	Married 76%	26-35 years 43.8%	Other 10.2%	Upper BA 4.2%	from 10 to 20 years 35.4 %
**Contracted 22.2%**			36-45 years 35.1%			More than 20 years 10.5%
**Official 30.8%**			46-55 years 6.2%			
			over 55 years 0.3%			

All cases were normally distributed except distributive justice. The correlation between nurses tends to leave with organizational justice were 23 percent, with distributive justice were 21 percent, and with procedural justice and interactional justice were 19 percent. All dimensions are inversely related (negatively) with turnover intentions with significant relationship (Pvalue=0.01) (Table 3)

**Table 3:** Descriptive statistics, Kolmogorov-Smirnov test results

	*The dimensions of organizational justice and turnover intentions*			*K-S test results*		*Associated with leaving the service*			
		
Variable	Number	Mean	± SD	Sig.	Statistics	Number	Spearman	Pearson	Sig.
Organizational Justice	344	2.59	0.04	0.524	0.812	162	-0.234**	-	0.01
Distributive justice	344	2.03	0.04	0.004	2.242	162	-	**-0.0217	0.01
Procedural justice	344	2.75	0.05	0.245	1.025	162	-0.191**	-	0.01
Interactional justice	344	2.81	0.05	0.098	1.225	162	-0.197**	-	0.01
Turnover	344	3.10	0.07	0.212	1.005	162	1	-	-

## 4. Discussion

The results showed that there is a weak negative relationship between organizational justice and a desire to leave in the nurses. Multiple regression analysis in 99% confidence level, showed a linear relationship between organizational justice and a desire to leave in the nurses. Other studies have shown that the results of this study are consistent. Handelan (2009) study concluded that organizational justice has a direct impact on leaving job (Handlon, 2009). Mac nob (2009) in his study showed that organizational justice is affected on leaving job through job satisfaction (McNabb, 2009). Holin (1991) and Schwarz Wald et al. (1992) stated that the lack of equality and justice associated with leaving work (Ryals & Knox, 2001; Schwarzwald, Koslowsky, & Shalit, 1992). Nedd (2006) found a significant relationship between structural empowerment and nurse turnover. Numerous studies have linked empowerment directly and indirectly to job satisfaction and commitment (Nedd, 2006).

Find the causes of this problem in a study in Taiwan in 2009, the most important factors related to turnover among nurses were willing to pay for job dissatisfaction, discontent in the transmission shifts and so on. The authors stated that these factors related on injustice. Nurses in comparison to other parts of the hospital and other hospitals were complaining of injustice and to prevent staff turnover hospital offered increased job satisfaction models to administrators (Ma, Lee, Yang, & Chang, 2008). The results of studies on the relationship between organizational justice and turnover showed this connection, in other words whenever organizational justice is increased, employees tends to stay is increased and relatively tends to job leaving is decreased.

The results showed that the weak negative relationship between procedural justice and a desire to leave the nurses is exist. The multiple regression showed that at 95% of Confidence level, there is a linear relationship between procedural justices and leaving the job in the nurses.

According to procedural theory whenever staff realize the justice of the current process of income distribution, have more incentive for better performance. The theory of procedural justice wants to knows the causes of fair or unfair perception of process and its effects (Moorman, 1991).

The results showed a weak negative relationship between distributive justice and a desire to leave in the nurses. The multiple regression showed that at 95% of Confidence level there is a relationship between distributive justices and tend to leave in the nurses. In the study Aghai et al., the results showed a significant negative correlation between the distribution justice and turnover intentions. The results of the regression analysis showed that distributive justice is a predictor of turnover of employees (Aghaei, Moshiri, & Shahrbanian, 2012). It is important to understand that distributive justice has great effect on organizational commitment and tending to leave the job. These findings have important implications for managers in developing appropriate strategies, policies and practices to improve employee organizational commitment and to reduce employee turnover intentions (Ponnu & Chuah, 2010). Distributive justice refers to fair judgment distribution of results, such as the distribution of promotion opportunities available in an organizational context (Cohen-Charash & Spector, 2001). Greenberg (1987) stated that perceived injustice of distributive justice makes people to feel injustice and as a result, they have lower productivity for organizations and they are less satisfied which ultimately leaving their work appears (Greenberg, 1987). Spencelaschinger et al. were shown that nurses perceptions of empowerment, supervisor incivility, and cynicism were strongly related to job satisfaction, organizational commitment, and turnover intentions that current research results confirm Heather et al research results (Spencelaschinger et al., 2009).

It is important to say that the findings of current research about the effects of distributive and procedural justice also consist with the Nadiri and Tanova (2010) research, they found that distributive justice tended to be a stronger predictor of all of the study variables compared to procedural justice. Their findings suggest that the fairness of personal outcomes that employees receive may have more impact on turnover intentions, job satisfaction and organizational citizenship behavior (OCB) than the perceived fairness of a firm’s procedures. It was also found that even though improved job satisfaction seems to be related to OCB, organizational justice seems to be the key factor that has a strong effect on both OCB and job satisfaction (Nadiri & Tanova, 2010). Another research in this issue was DeConinck and Stilwell (2004) research, the results showed that procedural justice was an important, direct predictor of supervisor satisfaction, while distributive justice predicted pay satisfaction. Both justice variables were only indirect predictors of organizational commitment. Role conflict was a significant predictor of organizational commitment both directly and indirectly through supervisor satisfaction, but role ambiguity was a predictor of only supervisor satisfaction and not organizational commitment. Pay satisfaction and supervisor satisfaction had a direct influence on withdrawal cognitions (DeConinck & Stilwell, 2004).

Interactional justice effects on individual aspects of the decision, especially equality in behavior of decision makers in decision-making processes. Personal behavior consists of trust in the relationship and behavior of individuals and humility and respect. Pearson correlation coefficient was used to assess the fourth hypothesis. The results showed that the weak negative relationship was between interactional justice and a desire to leave in the nurses. Multiple regression was used to indicate that on the 99% confidence level, there is relationship between interactional justices and leaving job in the nurses. The results of the study by Hosni and Jodet Kerdler showed that only about three percent interactional justice was affected on tending to turnover (Hsani & Jodat Kordlar, 2011). Another study showed that the most important predictors included; mental health, components of interactional justice, job commitment, procedural fairness, balance between work and life, finances and welfare issues, job security, and human relations in the workplace (Mostafavi Rad & Zahedi, 2011). In Aghai et al. research also there is significantly negative relationship between interactional justice and a desire to leave (Aghaei et al., 2012). The other researchers also found that burnout played an important role in predicting retention outcomes, explaining 4-12% of incremental variance in job satisfaction, organizational commitment, and turnover intentions (Abraham, 2000; Bernerh et al. 2007; Wanous et al, 2000). This research results also consist with Laschinger et al. researches, they shown that in nursing when work environments are structured in this way, nurses experience lower level of burnout, which, in turn, result in greater job satisfaction and fewer adverse patient events (Laschinger & Leiter, 2006; Leiter & Laschinger, 2006; Manojlovich & Laschinger, 2007).

Overal the results showed that “there is relationship between organizational justices and desire to turnover in nurses in hospitals of Urmia University of Medical Sciences”; In other words organizational justice and turnover intentions has inverse relationship, though whenever organizational justice is high, employees tend to stay in the organization increased or leaving job will decrease. This will bring positive consequences for the organization.

The impact of organizational justice dimensions on tending to leave job among nurses in different hospitals of Urmia University of Medical Sciences were in different levels. At the same time there is significant relation between the different dimensions of organizational justice and willingness to quit the job in nurses. The most important factor in nurses tends to leave was interactional justice variable and procedural justice and distributive justice variables were in the next rankings.

In general, to maintain and develop each component of organizational justice and also reduce the rate of turnover in nurses, the following steps are suggested:

(A) Distributive justice: efforts should be made that benefits, bonuses, financial incentives and promotions are fairly and equitably allocated among nurses for strengthen nurses’ perceptions of distributive justice, after identifying the perception of discrimination, appropriate measures to respond the people’s material and spiritual needs should be considered.
(B) Procedural justice: guidelines and procedures and decisions should be transparent.
(B) Interactional justice: the decisions taken on the organization should be available for the nurses in appropriate interaction and good manner.

Given the importance of organizational justice in the explanation and prediction of staff turnover recommended adequate training courses for managers and supervisors to make them familiar with the principles of organizational justice and its applications to reduce the amount of turnover among nurses in hospitals, to be held and managers and supervisors are encouraged to use advantages of this research in interaction with nurses.
